# A randomized, double-blind, placebo-controlled, hybrid parallel-arm study of low-dose naltrexone as an adjunctive anti-inflammatory treatment for major depressive disorder

**DOI:** 10.1186/s13063-022-06738-3

**Published:** 2022-09-30

**Authors:** Julia R. Plank, Stephanie C. Glover, Ben D. Moloney, Nicholas R. Hoeh, Frederick Sundram, Rachael L. Sumner, Suresh Muthukumaraswamy, Joanne C. Lin

**Affiliations:** 1 School of Pharmacy, Faculty of Medical and Health Sciences, University of Auckland, 85 Park Road, Grafton, Auckland, 1023 New Zealand; 2 Department of Psychological Medicine, School of Medicine, Faculty of Medical and Health Sciences, University of Auckland, 22-30 Park Avenue, Grafton, Auckland, 1023 New Zealand

## Abstract

**Background:**

Major depressive disorder (MDD) is a leading cause of disability worldwide. The current treatments are ineffective in approximately one-third of patients, resulting in a large economic burden and reduced quality of life for a significant proportion of the global population. There is considerable evidence that increased inflammation may distinguish a sub-type of MDD, and there are no validated diagnostic tools or treatments for neuroinflammation in MDD patients. The current study aims to explore the potential role of low-dose naltrexone (LDN), a drug with purported anti-inflammatory properties in the central nervous system, as an adjunctive treatment in patients with MDD.

**Methods/design:**

This double-blind placebo-controlled hybrid parallel arm study enables the exploration of peripheral and central inflammatory markers with LDN as an approach to investigate inflammation as a pathophysiological contributor to MDD. Eligible participants with MDD (*n* = 48) will be stratified into the high and low inflammatory groups according to the levels of high-sensitivity C-reactive protein (hs-CRP) and then randomized to receive LDN or placebo for an initial 12 weeks, followed by a further 12 weeks during which all participants will receive LDN. The primary outcome measure will be the Montgomery-Åsberg Depression Rating Scale (MADRS) administered at baseline, 2 weeks, 4 weeks, 8 weeks, 12 weeks, 14 weeks, 16 weeks, 20 weeks, and 24 weeks, to assess the effectiveness of the anti-depressant response. The secondary outcomes include the use of MRI techniques including quantitative magnetization transfer (qMT), echo-planar spectroscopic imaging (EPSI), and diffusion-weighted imaging (DWI) to help to elucidate the neurobiological mechanism of LDN, and the inflammatory mechanisms in action in MDD. Electroencephalography, blood samples, cognitive tasks, and additional questionnaires will also be used to determine if there is a specific profile of symptoms in individuals with inflammatory MDD. Healthy participants (*n* = 24) will be recruited for baseline outcome measures only, to enable comparison with patients with MDD.

**Discussion:**

This trial contributes to the literature on inflammation in MDD, including the understanding of the pathophysiology and efficacy of anti-inflammatory treatments. The investigation of inflammatory mechanisms in MDD is an important first step in the development of biomarkers to classify patient sub-groups, increase the accuracy of diagnosis, and tailor the approach to patients in clinical practice. This study may provide evidence of the benefit of LDN for the groups in whom conventional anti-depressants are ineffective and lead the way for translation into clinical practice.

**Trial registration:**

Australian New Zealand Clinical Trials Registry ACTRN12622000881730. Registered on 21 June 2022

**Supplementary Information:**

The online version contains supplementary material available at 10.1186/s13063-022-06738-3.

## Background

Approximately 280 million individuals suffer from depression worldwide, and depression is now the leading cause of disability [1]. Major depressive disorder (MDD), described as a persistent low mood for more than 2 weeks by the Diagnostic and Statistical Manual of Mental Disorders (DSM-V), can severely impact an individual’s ability to function [2]. Current treatments for MDD are ineffective in approximately one-third of patients resulting in poor outcomes, significant economic burden, and reduced quality of life for a significant proportion of the global population [3].

The current pharmacological therapies for MDD primarily target the monoaminergic systems, based on the theory that depression is due to a reduction in monoaminergic neurotransmission [4]. Conventional treatments, such as selective serotonin reuptake inhibitors (SSRIs), monoamine oxidase inhibitors (MAOIs), tricyclic antidepressants, and selective noradrenaline reuptake inhibitors (SNRIs), all target the monoaminergic system by various mechanisms. Recent research demonstrates that depression is influenced by factors beyond monoamines, such as neuroinflammation [5, 6].

Depression is a heterogenous disorder with varying pathophysiology, and substantial evidence suggests increased inflammation may distinguish a sub-type of MDD, present in 30% of total MDD cases [7]. Evidence of increased inflammation is demonstrated in MDD by (a) elevated peripheral levels of cytokines, chemokines, and circulating immune cells [5]; (b) post-mortem and microarray studies [8]; (c) that depression is a common comorbid disorder in neurodegenerative conditions with a known inflammatory component [9]; (d) sickness behavior, characterized by fatigue, anhedonia, and loss of appetite, is observed in both MDD and in cases of infection or inflammation [10, 11]; (e) MDD is often associated with factors which increase inflammatory markers such as stress, reduced sleep, and obesity [12]; and (f) medications that modulate the immune system, such as interferon, appear to affect mood [6]. Despite considerable evidence implicating inflammatory processes in MDD, there are no validated diagnostic tools or treatments for neuroinflammation in MDD.

Measurement of neuroinflammation in living patients is currently limited to two options. Lumbar puncture for the acquisition of cerebrospinal fluid is invasive and unable to identify the site of neuroinflammation [8]. Positron imaging tomography (PET) scanning utilizes ionizing radiation and is expensive, so it is not ideal for routine clinical use. Magnetic resonance imaging (MRI) is non-invasive, comparatively affordable, and more easily accessible. Advances in MRI techniques offer the possibility of measurement of neuroinflammation in people with MDD to better understand the pathophysiology of depression.

Quantitative magnetization transfer (qMT) and echo-planar spectroscopic imaging (EPSI) demonstrate sensitivity to changes in markers associated with inflammatory activity in the brain, such as water content of tissue [13] and brain temperature, respectively. qMT measures the exchange of magnetization between immobile protons bound to macromolecules, such as in myelin or membrane lipids, and the mobile protons in free water in intra- and extra-cellular tissue. qMT parameters, such as the forward exchange rate, which quantifies the efficiency of the magnetization transfer, are sensitive to the effects of neuroinflammation [14].

EPSI is a technique used for brain thermometry by measuring the chemical shift of the temperature-dependent water resonance frequency compared to a temperature-independent metabolite such as creatine [15, 16]. Brain temperature is expected to increase during neuroinflammation due to microglial activation, which increases metabolic demands leading to the release of excess heat. In a study of patients with chronic fatigue/myalgic encephalomyelitis, a condition believed to represent chronic low-level neuroinflammation, elevations of regional brain temperature between 0.28 and 0.50 °C were observed compared to control participants [17].

Diffusion-weighted imaging (DWI) techniques are used to investigate microstructural abnormalities in white matter. Recent advances in diffusion techniques show potential in detecting neuroinflammatory components such as astrogliosis and demyelination [18]. A novel diffusion technique named diffusion kurtosis imaging (DKI) has been used to indicate neuroinflammation in conditions such as traumatic brain injury [19], stroke [20], and multiple sclerosis [21]. qMT, EPSI, and DWI demonstrate promise for measurement of neuroinflammation in patients with MDD and will be used to (1) investigate potential biomarkers of brain inflammation in participants with MDD compared to control participants and (2) to monitor the neurobiological response to anti-inflammatory adjunctive treatment.

There is evidence to suggest that anti-inflammatory treatments have anti-depressant effects. However, the research does little to elucidate the mechanisms by which the anti-depressant effects occur [22]. Such studies have focused on the therapeutic efficacy of anti-inflammatory and immunomodulatory medications without clearly understanding the mechanism in action, thus leading to mixed results. Traditional non-steroidal anti-inflammatory drugs (NSAIDs), cytokine inhibitors, omega-3 fatty acids, and N-acetylcysteine are among the medications trialed for an anti-depressant response [23, 24]. There are critical limitations of these medications, however, including that they do not deeply penetrate the central nervous system tissue, are associated with adverse effects such as the increased risk of immunosuppression and stroke, and evidence suggests COX-2 selective NSAIDs may increase glial cell activation and neuroinflammation contrary to the anti-inflammatory hypothesis [25].

Low-dose naltrexone (LDN) is an atypical opioid antagonist with purported immunomodulatory and central anti-inflammatory effects [26]. Naltrexone is most often used for the treatment of opioid and alcohol addiction. However, the use of daily low doses (4.5 mg/day, 1/10th of the dose for addiction) appears to have anti-inflammatory effects in conditions such as Crohn’s disease [27, 28] and multiple sclerosis [28–30]. In addition to its role as a competitive opioid receptor antagonist, naltrexone also has an antagonist effect on non-opioid receptors, including Toll-like receptor-4 (TLR-4) found on microglia [31, 32]. Microglia activation results in sickness behaviors due to the release of inflammatory factors. By blocking TLR-4, the microglia may be prevented from assuming an inflammatory state, thus stopping the release of pro-inflammatory cytokines and neurotoxic superoxides [33]. Furthermore, naltrexone causes a continuous blockade of the opioid growth factor receptor axis (OGFr), resulting in the proliferation of immune cells [34, 35]. Though at the most common low dose of naltrexone, 4.5 mg/day, there is reduced proliferation of T and B cells due to an intermittent blockade.

In a small pilot trial of individuals with fibromyalgia, a significant reduction in several pro-inflammatory cytokines as well as improved mood was observed following treatment with LDN [36]. Furthermore, in a small trial of 12 individuals with MDD, LDN augmentation in addition to dopamine-enhancing agents was associated with reduced depressive symptomology [37]. LDN may be an effective adjunctive anti-inflammatory treatment for depressive symptoms in MDD.

The current study explores the potential role of LDN, a drug with purported anti-inflammatory properties in the central nervous system, as an adjunctive treatment in people with MDD. Moreover, using MRI techniques including qMT, EPSI, and DWI will help elucidate the neurobiological mechanism of LDN and the inflammatory mechanisms in action in MDD. This double-blind placebo-controlled hybrid parallel arm study enables the exploration of peripheral and central inflammatory markers with LDN as an approach investigating inflammation as a pathophysiological contributor to MDD.

## Methods/design

### Participants

The participants (*n* = 48) will be adults aged 18 to 55 years with moderate MDD currently receiving antidepressant medication. Healthy participants (*n* = 24) will be recruited to complete the baseline outcome measures to enable comparison with individuals without MDD. The complete inclusion criteria are outlined in Table 1. Strict exclusion criteria will be applied to limit the heterogeneity of the sample. The exclusion criteria are outlined in Table 2.

**Table 1:** Inclusion criteria

	Inclusion criteria
**All participants (*n*= 72)**	
Consent	Willing and able to give informed consent for participation in the trial
Demographics	
Age	18–55 years
Lifestyle considerations	Agreement to abstain from alcohol and drugs (except regular prescribed medication declared at screening) for 24 h prior to the start of each MRI and EEG session
Medication adherence	Ability and willingness to consume oral medication according to the prescribed dosing regimen
**Participants with MDD (*n*= 48)**	
Mental health	
Diagnosis	MDD according to the DSM-5 criteria
Symptomology	Moderate depression, ≥ 18 on the MADRS
Treatment status	Receiving treatment with an anti-depressant medication
Treatment plan	Plans to remain on the same anti-depressant medication for at least 12 weeks
Peripheral inflammation	Hs-CRP ≥ 3 mg/L or ≤1 mg/L

**Table 2:** Exclusion criteria

	Exclusion criteria
**All participants (*n*= 72)**	
Consent	Unable to give informed consent.
Safety	Contraindications to MRI, including refusal to be informed of an incidental finding during MRI examination.
Pregnancy	Pregnant or breastfeeding.
Health status	
Lifetime	Any bipolar disorder or psychiatric disorder or psychotic features as determined by the MINI. Anxiety disorders are not strictly excluded unless they are a primary cause of depressive symptoms.
	Neurological disorders or neurodegenerative conditions.
	Autoimmune disorders or chronic pain.
	Clinically significant medical conditions, e.g., seizure disorders, history of cancer.
Current	Acute risk of suicide as determined by clinician interview, MINI, and MADRS.
	Acute infectious pathology or chronic or acute inflammatory diseases.
	Significant renal, hepatic, or cardiovascular conditions.
	> Stage II antidepressant resistance as defined by Thase and Rush [38].
	Not clinically stable for ≥ 4 weeks.
Medication and drug status	Long-term anti-inflammatory or immunosuppressive therapy.
	Use of prescription opioid analgesics, antipsychotics, psychostimulants, and dopamine agonists.
	Substance use disorders within the last 12 months.
	Current recreational use of opioid-based drugs.
	Allergy or intolerance to naltrexone.
	Female participants of child-bearing age and not on a medically acceptable form of contraception.
**Healthy participants (*n*= 24)**	Any physical or psychiatric illness including major depressive disorder.
	Hs-CRP < 1 mg/L.
	Females on hormone suppressants.
	Current use of any medication except birth control.

### Participant recruitment

Participants will be recruited by the members of the study team from general practices within the greater Auckland area and via advertisements placed in local newspapers, noticeboards, and online using social media, allowing potential participants to make initial contact. Participants may also be recruited from an ethics-approved database of individuals who previously expressed interest in participating in studies on MDD (Auckland Health Research Ethics Committee number AH23223). Participants will be checked for eligibility at a screening visit and approved for inclusion in the trial by the study psychiatrist. The participant information sheet and informed consent form will be provided to participants prior to screening, allowing time for participants to seek independent advice. The participant information sheet and informed consent form provide details on the study including the type of trial, proposed involvement of participants, possible side effects, and risks of participation. Participants will have the opportunity to ask questions of the study investigators prior to and during the screening visit. If individuals choose to participate, verbal understanding of the information and written informed consent will be given to members of the study team at the screening.

### Study design

**Fig. 1:**
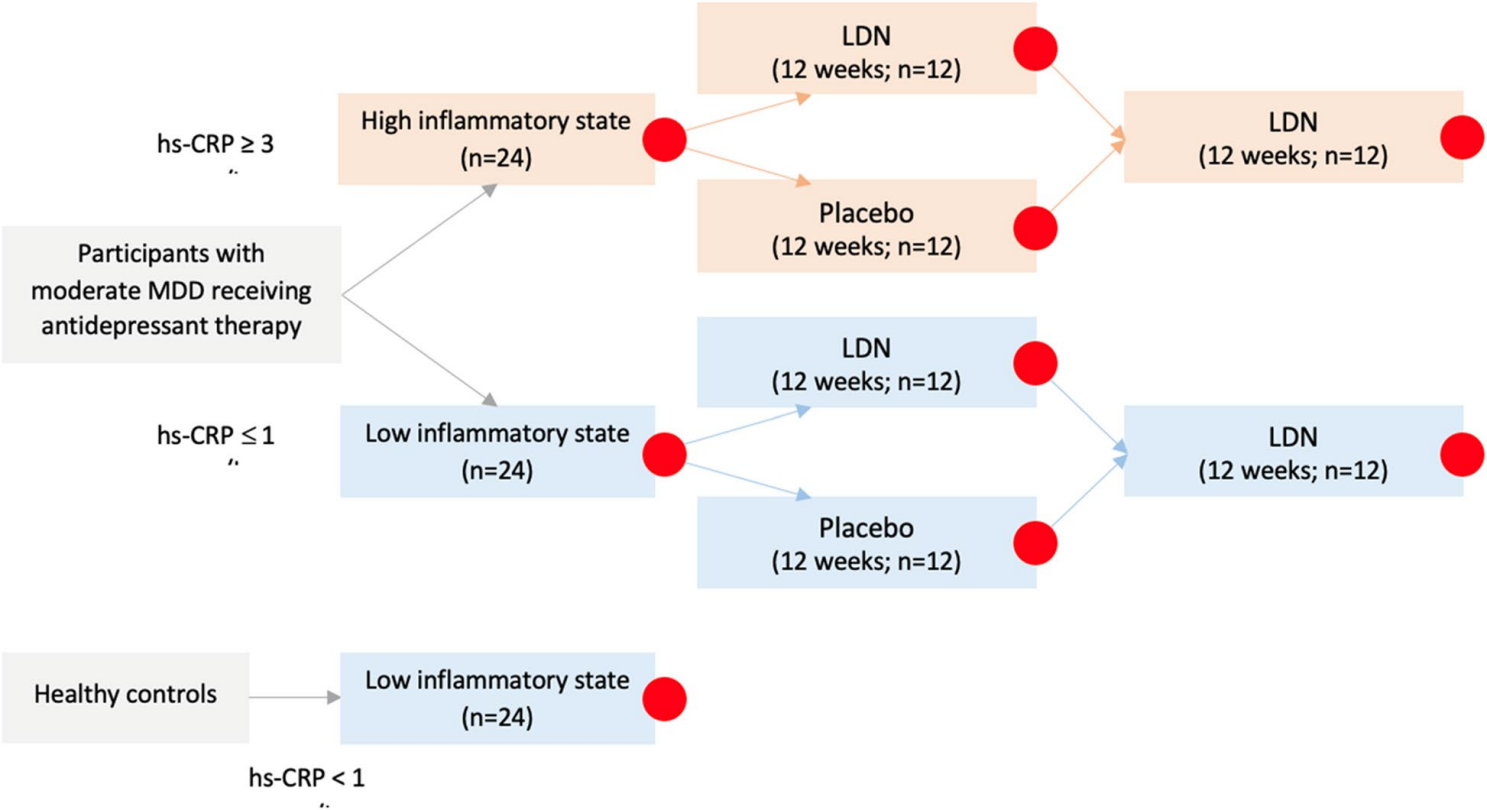
Flowchart of participant stratification based on the level of high-sensitivity C-reactive protein (hs-CRP). *Note*: circles denote the study sessions where primary, secondary, and tertiary outcomes will be measured

A randomized, double-blind, placebo-controlled, hybrid parallel-arm, superiority study (Fig. 1) will be used to test the potential of LDN as an adjunctive treatment for MDD. Participants with MDD will be allocated into parallel groups of high and low inflammatory status in blocks of 12 in a 1:1 ratio. The study will take place primarily at the Clinical Research Centre at the University of Auckland. After an initial screening, participants with MDD who meet the inclusion criteria will be prospectively stratified into low/high inflammatory status (*n* = 24 per group) based on the levels of high-sensitivity C-reactive protein (hs-CRP). Hs-CRP is a pro-inflammatory acute phase protein. Serum chemistry and hematology will also be conducted at the initial screening, including measurements of complete blood count, liver function tests, hs-CRP, and a human chorionic gonadotrophin (hCG) pregnancy test. The laboratory variable stated normal ranges will be used to categorize the results, and an additional interpretation by the study’s clinicians will deem abnormal values as either clinically or not clinically significant. A second blood sample, measuring only hs-CRP, will be taken at least 1 week later and be used to confirm inflammatory status. A high inflammatory state will be characterized by hs-CRP ≥ 3 mg/L, and a low inflammatory state will be characterized by hs-CRP ≤ 1 mg/L. Once the inflammatory status is confirmed, participants will complete a urine drug test and their baseline assessments and be randomly assigned to receive either placebo or LDN.

The primary, secondary, and tertiary endpoints of the study will be measured at baseline, following 12 weeks of LDN or placebo and following a further 12 weeks of LDN only. Previous studies using LDN indicate that a minimum of 12 weeks is required to observe anti-inflammatory effects [26, 39]. The anti-depressant effects of LDN will be measured using the Montgomery-Åsberg Depression Rating Scale (MADRS) which assesses the severity of depressive symptoms [40].

The primary outcome for this trial is change in MADRS scores at 12 weeks relative to baseline. The secondary outcomes include change in MADRS scores at 12 weeks in the high inflammatory group versus the low inflammatory group, detection of central inflammation in MRI scans, and measurement of peripheral inflammatory markers.

MRI scans will be conducted using the 3-T Siemens Vida Fit scanner at the Centre for Advanced MRI at the University of Auckland. A T1-weighted image will be acquired for segmentation and anatomical reference using a magnetization prepared rapid gradient echo (MPRAGE) sequence: repetition time (TR) = 2000 ms; echo time (TE)= 2.85 ms; flip angle= 8^o^; 208 slices; slice thickness= 1.00 mm, field of view (FOV) = 256 × 256 mm, matrix = 256 × 256, voxel size = 1.0 × 1.0 × 1.0 mm, and acquisition time (TA) = 4 min 56 s.

The qMT technique will be used to measure the magnetization exchange rate. To acquire the qMT data, a series of MT-weighted 3D fast low-angle shot (FLASH) sequences are applied: echo time (TE) = 3.78 ms, repetition time (TR) = 35 ms, voxel size = 2 × 2 × 5 mm, varying flip angles for T1 mapping, and acquisition time (TA) = 10 min. Four pulse powers of 1000 degrees, 900 degrees, 800 degrees, and 400 degrees are applied to acquire 2, 3, 1, and 2 measurements, respectively. Finally, FABBER CEST in FSL is used to model the MT parameters.

Brain temperature will be calculated from the whole-brain spectroscopy data obtained using the EPSI sequence: TR1 = 1710 ms, TR2 = 591 ms, TE = 17.6 ms, voxel size = 5.6 × 5.6 × 10 mm, and TA = 17 min. The Metabolite Imaging and Data Analysis System (MIDAS) package will be used to analyze the data. The formula *T*_brain_ =  − 102.61 × ∆_water − CRE_ + 206.1^°^*C* will be used to calculate the absolute brain temperatures according to the distance between the creatine (CRE) and water peaks.

DWI scans will use a three-shell protocol to acquire 18 non-diffusion-weighted images (*b* = 0 s/mm^2^) and 180 diffusion-weighted images (30 at *b* = 500 s/mm^2^; 60 at *b* = 1000 s/mm^2^; 90 at *b* = 2000 s/mm^2^) using non-collinear diffusion-weighting directions. The following are the other imaging parameters: TE/TR = 78/12,500 ms, slice thickness 2 mm, FOV = 224 × 224 mm, matrix = 112 × 112, resulting in 2 mm^3^ isotropic voxels, and TA = 15 min.

Blood samples will be acquired to measure peripheral inflammatory markers: hs-CRP, interleukin (IL)-1β, IL-2, IL-6, and IL-8. IL-10, IL-12p70, tumor-necrosis factor (TNF)-α, interferon-inducible protein (IP)-10, monocyte chemoattractant protein (MCP)-1, vascular endothelial growth factor (VEGF), regulated upon activation, normal T cell expressed, secreted (RANTES), erythrocyte sedimentation rate (ESR), and glycoprotein nonmetastatic melanoma protein B (GPNMB) will be analyzed using multiplexed bead-based immunoassays. The blood samples will be analyzed for ESR 1 h after collection. Prior to analysis of the remaining peripheral inflammatory markers, the blood samples will be centrifuged, and the plasma will be frozen at − 80 °C.

Tertiary outcomes include electroencephalography (EEG) assessments and questionnaires compared between baseline, at 12 weeks, and at 24 weeks and the change in MADRS score between 12 and 24 weeks. Questionnaires for tertiary outcomes include the Beck Depression Inventory (BDI-II; [41]), Behavioural Activation for Depression Scale (BADS; [42]), Profile of Mood States (POMS; [43]), Lifetime Stress and Adversity Inventory (STRAIN; [44]), the SF-36 health survey [45], and the Sickness Questionnaire (SicknessQ; [46]). These questionnaires will enable a thorough understanding of each participant’s symptoms, stressors, and quality of life. The National Institute of Health (NIH) Toolbox will be used to assess the aspects of cognition including attention, executive function, psychomotor speed, and memory [47]. Resting-state and task-based electroencephalography (EEG) scans will be conducted with tasks including doors [48], long-term potentiation (LTP, [49]), and the attention network task (ANT; [50]) to assess responsiveness to positive and negative feedback, memory, and attention. In combination, these outcomes will be used to determine if a specific profile of symptoms is associated with inflammatory MDD.

Finally, the Generic Assessment of Side Effects (GASE, [51]) questionnaire will be used to monitor the adverse effects of LDN, and treatment expectancy effects will be measured using the Stanford Expectations of Treatment Scale (SETS, [52]). Pregnancy status will be reassessed at 12 weeks with a human chorionic gonadotropin (hCG) urine test.

**Fig. 2:**
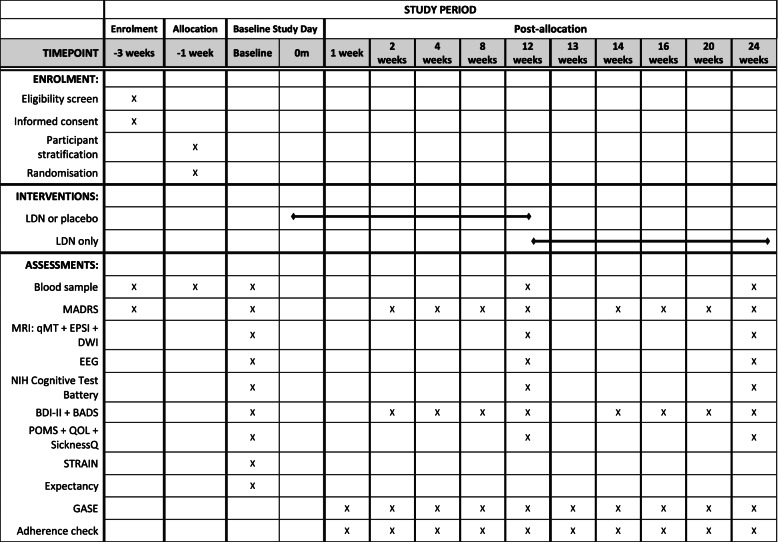
Standard Protocol Items: Recommendations for Interventional Trials (SPIRIT)

A complete list of outcome measures is summarized in the Standard Protocol Items: Recommendations for Interventional Trials (SPIRIT) figure (Fig. 2), and the SPIRIT checklist is provided as Additional file 1.

### Randomization, blinding, and code-breaking

Following stratification of the eligible participants into high inflammatory and low inflammatory status, participants will be randomly allocated to parallel LDN or placebo groups in blocks of 12 in a 1:1 ratio. Computer-generated randomization will be performed by one member of the research team. LDN or placebo will be prescribed by the study psychiatrist(s) and delivered to the participant by the study pharmacist(s). The randomizer and pharmacist will be the only members of the research team unblinded to the identity of the medication. To maintain triple-blind conditions, the participant and study team members conducting the measurements of primary and secondary outcomes will not be aware of the identity of the medication.

During patient debriefing, participants will be asked to identify the medication they think they received. When all participants in their randomization block have completed the trial, participants and study team members will be informed of the correct medication identity. If the participant experiences a reaction or acute deterioration of health during the study and requires medical intervention, the identity of the medication may be revealed. An on-call member of the study team will be available 24/7 to break blinding. While this member is blinded, the randomizer will have prepared code-break envelope which may be broken in the event unblinding is required.

### Drug preparation and administration

Naltrexone will be supplied to CompoundLabs by a licensed pharmaceutical material supplier who will confirm the identity and potency of the naltrexone. LDN capsules will be prepared by blending 1.5 mg naltrexone hydrochloride with microcrystalline cellulose (MCC) and filling the powder into a gelatin capsule shell. Placebo will be MCC only in a gelatin capsule shell. An unblinded pharmacist independent of the study team will store and dispense the interventions to participants, in accordance with Medicine Regulations 1984 [53]. The intervention will be dispensed to participants once per month. Participants in the durability arm will take one capsule orally once daily at night for 1 week, then 2 capsules at night for 1 week, then 3 capsules (4.5 mg) at night for 10 weeks. This dosing regimen will be repeated at 12 weeks, at the beginning of the 12-week open-label extension. If participants experience adverse effects, the dose may be titrated down to either 2 capsules daily or 1 capsule twice daily.

### Alterations to allocated interventions

There will be no special criteria for modifying or discontinuing the allocated medication.

### Strategies to improve adherence

Participants will be contacted by the study team after the day of medication delivery at 1 day, 3 days, 1 week, 2 weeks, 4 weeks, 6 weeks, 8 weeks, 10 weeks, 12 weeks, 13 weeks, 14 weeks, 16 weeks, and 20 weeks. The study team may contact participants via email, phone call, or text message to remind participants to adhere to study protocols (e.g., take medication daily) and to check for any adverse effects. At 4, 8, 16, and 20 weeks, participants will return to the University of Auckland for a treatment refill. At 12 and 24 weeks, participants will return to the University of Auckland for follow-up measurements of all outcomes. Participants will return their capsule bottles at the end of each 4-week period, and the number of capsules remaining in the bottle will be used to calculate intervention compliance.

### Relevant concomitant care and post-trial care

There will not be any modification to the usual access to care pathways while participants are enrolled in the study. If a participant becomes ineligible for the study while enrolled, the participant may be discharged from the trial. If a participant is discharged due to physical health or psychiatric reasons, the participant will be referred to appropriate medical services. Upon completion of the study, participants will be reimbursed for their time in grocery vouchers. Individuals who complete initial blood tests but do not meet the inclusion criteria will be given a $20 grocery voucher. Healthy control participants who complete a single visit will be given $80 in grocery vouchers. Individuals with MDD who participate in the 24-week trial will receive a total of $320 in grocery vouchers: $80 at each study visit and $20 at each collection of their next treatment supply. There is no expectation of harm to participants caused by the current trial.

### Statistical analyses and power calculations

Linear mixed effects models will be used to assess the primary outcome (change in MADRS scores at 12 weeks). Time (baseline and at 12 weeks) and drug (naltrexone and placebo) will be treated as fixed effects, and participants will be treated as a random effect. The primary estimand of interest will be the time × drug interaction coefficient with associated confidence intervals. Satterthwaite’s method (two-tailed) will be used to obtain *p*-values with an alpha set at *p* < .05.

To inform the power calculations for the primary outcome, a sensitivity analysis of the primary outcome was conducted for the fixed sample size of 48 (12 participants per group). Monte Carlo simulations were conducted using the mixed effects model described above with 10,000 simulations per run. Data were simulated on each iteration using the following parameters: *n* = 48, 4 dropouts with data missing at random, *α* = 0.05, (1−β) = 0.8, baseline MADRS scores of 30, random effect variance = 6.32, and error variance = 4.89. Variance estimates were obtained from linear mixed effects models fit to data from a previous antidepressant trial [54]. The present study is sensitive to detect changes of ~ 6 MADRS points according to the Monte Carlo simulations. The previous pilot study of 12 participants with MDD showed an 18-point decrease in MADRS scores with LDN compared to an 8-point drop with placebo [37]. The current study of 48 participants is considerably better powered for the primary outcome measure.

### Sub-group data analyses and missing data

Sub-group analyses, such as a comparison of the hs-CRP level between sexes, may be conducted if a significant effect on hs-CRP is observed. A modified intention-to-treat scheme will be used including all participants who received at least one dose of intervention. All missing data will be classified as “missing at random” or “not missing at random” prior to unblinding. Data imputation techniques will be used where necessary.

### Adverse event reporting

The GASE scale, a 36-item scale that assesses the most frequent side effects in clinical trials of medicines [51], will be used to evaluate the side effects at monthly visits. Any adverse events that occur during the trial will be recorded, and any serious adverse events will be reported within 24 h to the Centre for Adverse Reactions Monitoring consistent with the guidelines provided by the New Zealand Medicines and Medical Devices Safety Authority (“MedSafe”).

### Data and safety monitoring committee

The study will be conducted to International Conference on Harmonisation (ICH) Good Clinical Practice (GCP) clinical trial standards. Two independent consultant psychiatrists and a biostatistician will form the Data and Safety Monitoring Committee for the trial. The study is considered low risk given the comprehensive inclusion/exclusion criteria; however, in the unlikely event of a serious adverse event, the Safety Monitoring Committee may decide to suspend the study or request suspension until study protocols are appropriately revised.

### Data management and record keeping

Separate paper-based files will be kept for each participant; however, the majority of data will be captured by the online Research Electronic Data Capture (REDCap) tools hosted at the University of Auckland [55]. REDCap is a secure web-based platform designed to capture data and manage databases online. Demographics, medical history, height, weight, current medications, MADRS, and GASE scores will be entered directly into REDCap. Data from MRI scans and blood sample analysis (including hs-CRP and peripheral cytokine analysis) will also be stored electronically on secure University of Auckland servers on password-protected files.

During the study, participants will be identified by a unique study number and/or code. The name and any other identifying detail will not be included in any trial data electronic file. On all study-specific documents, other than the signed consent form, the participant will be referred to by their unique number/code. All data will be held for a period of 10 years from the completion of the study.

### Dissemination policy

The results from the present study will be published in selected academic journals and presented at academic conferences. The results may also be distributed through social media, community forums, or news outlets. Participants may request a summary of their individual results at the end of the trial.

## Discussion

The present study will investigate a novel anti-inflammatory drug as an adjunctive treatment for patients with MDD. LDN is already used off-label as an emerging therapy but is currently not prescribed in standard clinical practice. This study may provide evidence of the benefit of naltrexone for patient groups in whom conventional anti-depressants are ineffective and lead the way for translation into clinical practice.

The study’s unique design whereby participants are stratified into high/low inflammatory status may significantly contribute to the understanding of the pathophysiology of MDD. Despite evidence of inflammatory mechanisms in 30% of participants with MDD, diagnostic tools, and treatments for these patients are so far unclear: previous studies of anti-inflammatories have relied on heterogeneous groups of participants that led to mixed results. The present study will provide evidence on the efficacy of an adjunctive anti-inflammatory treatment specifically for patients with MDD associated with inflammation and enable comparison with a placebo treatment with patients of low-inflammatory status and with patients without MDD.

Finally, exploring potential blood-based and imaging biomarkers of peripheral and central inflammation may enable the development of more accurate diagnostic tools for patients with MDD and other disorders associated with inflammatory mechanisms. Inflammation is observed in various neuropsychiatric and neurological disorders, but the role of the inflammatory mechanisms in these conditions is unclear. Investigating inflammatory mechanisms in MDD is a critical first step in identifying biomarkers to classify patient sub-groups, increase the accuracy of diagnosis, and tailor the approach to patients in clinical practice.

## Trial status

The study will advertise for enrollment in September 2022. Enrollment is expected to be completed in July 2024.

## Supplementary Information

**Additional file 1.** SPIRIT Checklist.

## Data Availability

The datasets analyzed during the current study and statistical code are available from the corresponding author on reasonable request, as is the full protocol.

## Abbreviations

ANOVA: Analysis of variance
ANT: Attention Network Task
BADS: Behavioral Activation for Depression Scale
BDI-II: Beck Depression Inventory II
CRE: Creatine
Hs-CRP: High-sensitivity C-reactive protein
EEG: Electroencephalography
EPSI: Echo planar spectroscopic imaging
ESR: Erythrocyte sedimentation rate
DBSI: Diffusion basis spectrum imaging
DWI: Diffusion-weighted imaging
GASE: General assessment of side effects
GCP: Good Clinical Practice
GPNMB: Glycoprotein nonmetastatic melanoma protein B
HCG: Human chorionic gonadotropin
ICH: International Conference on Harmonisation
IL: Interleukin
IP-10: Interferon-inducible protein 10
LDN: Low-dose naltrexone
LTP: Long-term potentiation
MADRS: Montgomery-Åsberg Depression Scale
MCP-1: Monocyte chemoattractant protein-1
MDD: Major depressive disorder
MIDAS: Metabolite Imaging and Data Analysis System
MRI: Magnetic resonance imaging
MT: Magnetization transfer
NIH: National Institute of Health
NSAIDs: Non-steroidal anti-inflammatory drugs
POMS: Profile of Mood States
qMT: Quantitative magnetization transfer
RANTES: Regulated upon activation, normal T cell expressed, and secreted
REDCap: Research Electronic Data Capture
SETS: Stanford Expectations of Treatment Scale (SETS)
SNRI: Selective noradrenaline reuptake inhibitor
SSRI: Selective serotonin reuptake inhibitor
STRAIN: Lifetime Stress and Adversity Inventory
TA: Acquisition time
TE: Echo time
TR: Repetition time
TLR-4: Toll-like receptor-4
TNF-α: Tumor necrosis factor-α
VEGF: Vascular endothelial growth factor
